# Wild sympatric rodents inhabiting pig farm environments may facilitate the spillover of *Enterocytozoon bieneusi* from pig farms[Fn FN1]

**DOI:** 10.1051/parasite/2024061

**Published:** 2024-09-27

**Authors:** Fa Shan, Qingda Meng, Fang Wang, Jinfeng Zhao, Huiyan Xu, Nanhao Wang, Yufeng Liu, Sumei Zhang, Guanghui Zhao, Longxian Zhang

**Affiliations:** 1 College of Veterinary Medicine, Northwest A&F University Yangling 712100 PR China; 2 College of Veterinary Medicine, Henan Agricultural University Zhengzhou 450046 PR China; 3 Key Laboratory of Quality and Safety Control of Poultry Products (Zhengzhou), Ministry of Agriculture and Rural Affairs Zhengzhou 450046 PR China; 4 International Joint Research Laboratory for Zoonotic Diseases of Henan Zhengzhou 450046 PR China

**Keywords:** *Enterocytozoon bieneusi*, Rodent, Genotype, Pig farm, Zoonotic transmission

## Abstract

*Enterocytozoon bieneusi* is a zoonotic pathogen prevalent in mammalian and avian hosts across the globe. Wild small mammals, being abundant worldwide, serve as important sources of zoonotic disease transmission to humans. Here, 227 fecal samples were collected from five rodent and shrew species on 34 pig farms in China to investigate the prevalence and molecular characterization of *E. bieneusi*. The overall prevalence of *E. bieneusi* was 17.18% (39/227), with a distribution of 23.53% (32/136) in *Rattus tanezumi*, 8.62% (5/58) in *Rattus norvegicus*, and 8.00% (2/25) in *Mus musculus*. Eight *E. bieneusi* genotypes were identified, comprising four known genotypes: D (*n* = 8), EbpC (*n* = 8), PigEBITS7 (*n* = 9), and EbpA (*n* = 2), and four novel genotypes: CHPR1 (*n* = 7), CHPR2 (*n* = 1), CHPR3 (*n* = 2), and CHPR4 (*n* = 2). This study is the first to report *E. bieneusi* in rodents from pig farms in Henan, Shaanxi, and Shanxi Provinces in China. The host range of genotype EbpC was expanded with its first detection in *M. musculus* and *R. tanezumi*. All identified *E. bieneusi* genotypes belong to group 1, raising concerns about these sympatric rodents being reservoirs of zoonotic transmission. Moreover, the widespread distribution of genotype EbpC suggests potential cross-species transmission between sympatric rodents and domestic pigs. Our findings highlight the potential role of sympatric rodents in facilitating the spillover of *E. bieneusi* from pig farms, which could pose a potential public health threat.

## Abstract (fr)

*Enterocytozoon bieneusi* est un pathogène zoonotique répandu mondialement chez les hôtes mammifères et aviaires. Les petits mammifères sauvages, abondants dans le monde entier, constituent d’importantes sources de transmission de maladies zoonotiques à l’homme. Ici, 227 échantillons fécaux ont été collectés auprès de cinq espèces de rongeurs et de musaraignes dans 34 élevages porcins en Chine pour étudier la prévalence et la caractérisation moléculaire d’*E. bieneusi*. La prévalence globale d’*E. bieneusi* était de 17,18 % (39/227), avec une distribution de 23,53 % (32/136) chez *Rattus tanezumi*, 8,62 % (5/58) chez *Rattus norvegicus* et 8,00 % (2/25) chez *Mus musculus*. Huit génotypes d’*E. bieneusi* ont été identifiés, dont quatre génotypes connus (D (*n* = 8), EbpC (*n* = 8), PigEBITS7 (*n* = 9) et EbpA (*n* = 2)) et quatre génotypes nouveaux (CHPR1 (*n* = 7), CHPR2 (*n* = 1), CHPR3 (*n* = 2) et CHPR4 (*n* = 2)). Cette étude est la première à signaler la présence d’*E. bieneusi* chez des rongeurs provenant d’élevages porcins des provinces du Henan, du Shaanxi et du Shanxi en Chine. La gamme d’hôtes du génotype EbpC a été élargie avec sa première détection chez *M. musculus* et *R. tanezumi*. Tous les génotypes d’*E. bieneusi* identifiés appartiennent au groupe 1, ce qui soulève des inquiétudes quant au fait que ces rongeurs sympatriques soient des réservoirs de transmission zoonotique. De plus, la large distribution du génotype EbpC suggère une transmission interspécifique potentielle entre les rongeurs sympatriques et les porcs domestiques. Nos résultats soulignent le rôle potentiel des rongeurs sympatriques dans la facilitation de la propagation d’*E. bieneusi* à partir des élevages porcins, ce qui pourrait constituer une menace potentielle pour la santé publique.

## Introduction

Microsporidiosis is an emerging opportunistic disease caused by microsporidia infection, affecting both invertebrates and vertebrates, including humans [20, 38]. Microsporidia constitute a vast group of parasitic eukaryotes that live inside host cells, with over 1500 species distributed among 200 different genera, of which 17 species in 10 genera have been identified as agents of human infections [28, 38]. The predominant culprit of human microsporidiosis is *Enterocytozoon bieneusi*, which accounts for over 90% of documented cases [20, 28, 38]. Besides humans, *E. bieneusi* is widely present in mammalian and avian hosts worldwide, raising concerns about the role of animal hosts in the spread of the pathogen [37]. The most likely routes of *E. bieneusi* transmission to humans or animals are transmission through contact with infected hosts and ingestion of spores of environmental origin [43]. Indeed, in addition to direct infection of humans, infected hosts can release *E. bieneusi* spores into the environment, causing contamination of agricultural products and water sources. Consequently, there have been cases of foodborne and waterborne transmission of *E. bieneusi* [5, 7, 11, 46].

Due to their tiny size of about 1 μm, the spores of *E. bieneusi* can be mistaken for food particles, fungi, bacteria, and other microsporidia, posing a challenge for accurate diagnosis using conventional microscopy techniques. The ability to correctly identify *E. bieneusi* relies heavily on the skills of the examiner [37, 50]. While immunodetection with monoclonal antibodies is convenient for broad epidemiological studies, it does not provide genotyping information [1, 55]. However, the advent of molecular technologies, such as polymerase chain reaction (PCR) and internal transcribed spacer (ITS) DNA sequencing of ribosomes, has allowed for the global characterization of the molecular signature of *E. bieneusi* in humans, various domestic and wild animals, and aquatic environments [20, 23, 37]. As of now, over 500 *E. bieneusi* genotypes have been identified worldwide [20]. These genotypes have been classified into 11 groups (groups 1–11) by phylogenetic analysis [20, 23]. Genotypes in groups 1 and 2 have been found in a wide range of hosts, including humans, and are likely responsible for most zoonotic or cross-species *E. bieneusi* infections, whereas host-adapted genotypes appear to be more common in groups 3–11 [20, 22, 23]. Conducting molecular epidemiological studies is essential for genotyping *E. bieneusi* strains extracted from underrepresented animal hosts to gain a better understanding *E. bieneusi* epidemiology and assess the role of animals in its transmission to humans.

Rodents and shrews are widely distributed throughout the world, often gathering in dense concentrations, and come into close contact with humans and domestic animals [2, 17]. This proximity poses a potential threat to public health as they can destroy and contaminate food and transmit various pathogens, particularly in agricultural settings where food is abundant [30, 33]. Research has shown that there are about 60 different genotypes of *E. bieneusi* in rodents, with 18 of them being capable of zoonotic transmission, affirming the part that rodents play in spreading *E. bieneusi* [40, 53]. Pigs are highly susceptible to *E. bieneusi* infection and primarily harbor group 1 genotypes; thus, they are primary hosts for pathogenic strains that affect humans. Among these, the zoonotic genotypes EbpC, EbpA, O, H, and D are the most commonly found [23, 41]. In swine farm environments, there is a significant presence of wild small mammals, including rodents and shrews, which share the same living area and can come into close contact with humans and domestic pigs [2]. Therefore, it is essential to identify the epidemiological characteristics of *E. bieneusi* in these animals inhabiting swine farm environments and their role in transmission to prevent its spread among humans and other animals. Our research aimed to determine the molecular prevalence of *E. bieneusi* in rodents and shrews within swine farm environments in China by using nested PCR and sequence analysis of the ribosomal ITS region. Furthermore, the zoonotic potential of the isolates and possible routes of transmission of *E. bieneusi* were assessed through genotypic identification and phylogenetic analysis.

## Materials and methods

### Ethical standards

The research procedure was approved by the Research Ethics Committee of Henan Agricultural University in accordance with the Chinese Laboratory Animal Administration Act of 1998. The study team obtained permission from the farm owners or managers to access the farms and collect samples.

### Specimen collection

**Figure 1:**
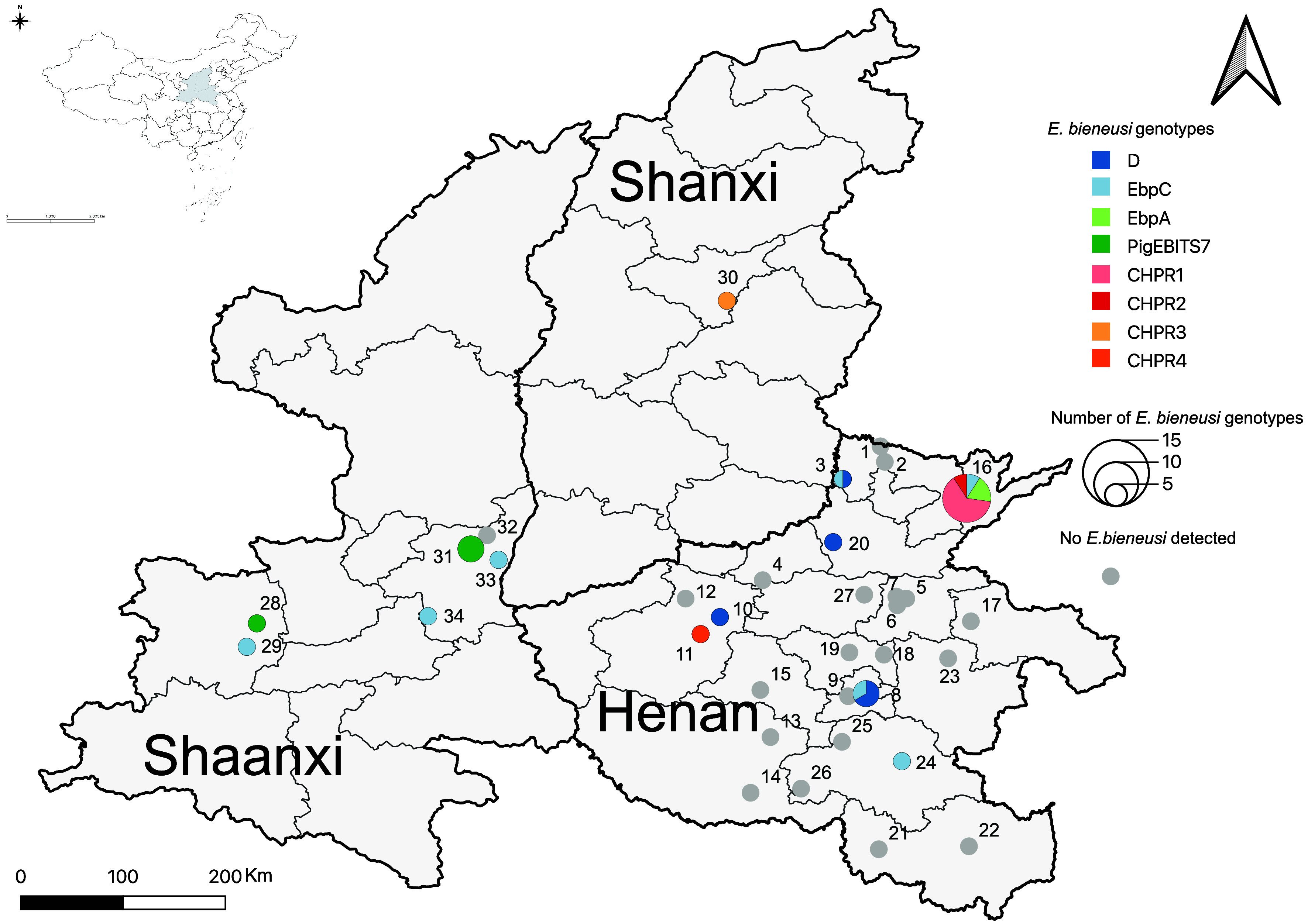
Map of the sampling areas and the geographical distribution of *Enterocytozoon bieneusi* detected in the present study. The sampled pig farms are represented by circles on the map and the numbers next to the circles represent farm numbers. The numbers of *E. bieneusi* genotypes on the surveyed pig farms are indicated by colored circles of different sizes.

Wild small mammal trapping was conducted between March 2021 and June 2023 on 34 pig farms situated in 18 cities across Henan, Shaanxi, and Shanxi Provinces in China (Fig. 1). Trapping cages were used for capture and were deployed on pig farms 1 h before dusk and collected within 1 h after sunrise. The trapping activities were performed for 3–5 days on each farm. In total, 227 wild small mammals (136 *Rattus tanezumi*, 58 *Rattus norvegicus*, 25 *Mus musculus*, one *Apodemus agrarius*, and seven *Crocidura shantungensis*) were captured (Table 1), with the number of captures ranging from 1 to 23 per farm. After capture, the wild small mammals were humanely euthanized via carbon dioxide inhalation. Each animal was then individually placed in labeled bags containing information that included sex, body weight, sampling date, sampling location, farm size, farm type, and duration of pig rearing. Within 48 h, the specimens were transported in containers with ice packs to the laboratory for necropsy, which was conducted in a biosafety cabinet. Fecal and liver samples were collected from each specimen and stored at −80 °C for subsequent analysis.

**Table 1:** Prevalence and genotypes of *Enterocytozoon bieneusi* among rodents and shrews inhabiting pig farms in China.

Host species	No. tested	No. positive (%)	Genotypes (n)
Asian house rat	136	32 (23.5%)	D (5), PigEBITS7 (9), EbpC (4), EbpA (2), CHPR1 (7), CHPR2 (1), CHPR3 (2), CHPR4 (2)
(*Rattus tanezumi*)			
Brown rat	58	5 (8.6%)	D (3), EbpC (2)
(*Rattus norvegicus*)			
House mouse	25	2 (8.0%)	EbpC (2)
(*Mus musculus*)			
Striped field mouse	1	0 (0%)	na
(*Apodemus agrarius*)			
Asian lesser white-toothed shrew	7	0 (0%)	na
(*Crocidura shantungensis*)			
Total	227	39 (17.2%)	D (8), PigEBITS7 (9), EbpC (8), EbpA (2), CHPR1 (7), CHPR2 (1), CHPR3 (2), CHPR4 (2)

n, number of specimens; na, not applicable.

### DNA extraction

A QIAamp PowerFecal Pro DNA kit (QIAGEN, Hilden, Germany) was used to extract the genomic DNA from each fecal sample (approximately 200 mg), according to the manufacturer’s instructions. The extracted DNA was stored at −20 °C for subsequent PCR analysis.

### Molecular analysis

Samples were screened for the presence of *E. bieneusi* by amplifying a 389-bp nucleotide fragment of the ITS segment using a previously described nested PCR method [39]. The negative controls for the experiment included double-distilled water, while the positive controls consisted of known DNA from cows with genotype D. Following amplification, the PCR products were separated by electrophoresis on 1% agarose gels containing DNAGREEN dye (Tiandz, Inc., Beijing, China). Positive samples were subjected to bidirectional sequencing at SinoGenoMax Biotechnology in Beijing, China. The obtained raw sequences in this study were then assembled and corrected using DNASTAR 7.1.0 (http://www.dnastar.com/) and aligned with reference sequences downloaded from GenBank. Genotypes were labeled according to the established nomenclature of the *E. bieneusi* 243-bp ITS region. A neighbor-joining phylogenetic tree was constructed using Mega 11 software with the Kimura-2 parametric algorithm and 1000 replicates to reveal the evolutionary relationships and zoonotic potential among the genotypes of *E. bieneusi* isolates.

### Statistical analysis

IBM SPSS version 27.0 (IBM Corp., Armonk, NY, USA) was used to conduct the statistical analysis. The risk factors associated with *E. bieneusi* infections were evaluated by calculating the odds ratio and 95% confidence interval (CI) through either univariate analyses (Chi-squared test or Fisher’s exact test) and multivariate analyses (Binary logistic regression analyses). The variables with *p* < 0.20 in the univariate analysis were introduced in the multivariate analyses. Statistical significance was determined at a *p*-value threshold of <0.05.

### Nucleotide sequence accession numbers

The unique nucleotide sequences obtained were submitted to the GenBank database under accession numbers PP158567–PP158577.

## Results

### Prevalence of *Enterocytozoon bieneusi*

In 39 out of 227 fecal samples, *Enterocytozoon bieneusi* was found, giving a prevalence rate of 17.2% (95% CI, 12.2–22.1). Among the infected individuals, there were 32 *R. tanezumi* (23.5%, 16.3–30.7), five *R. norvegicus* (8.6%, 1.2–16.1), and two *M. musculus* (8.0%, −3.4–19.4) (Table 1). A statistically significant difference was observed between these groups (*χ*^2^ = 8.029, *p* = 0.018) (Table 2). However, all individuals of *A. agrarius* and *C. shantungensis* tested negative for *E. bieneusi*. Regarding regional distribution, a statistically significant difference was found in the prevalence of *E. bieneusi* infection among the three provinces included in this study (*χ*^2^ = 16.795, *p* = 0.002). The prevalence rates of *E. bieneusi* infection in Shanxi, Shaanxi, and Henan were 50.0% (2/4), 35.3% (12/34), and 13.2% (25/189), respectively (Table 2). To ensure statistical accuracy, data from Shanxi Province were excluded from the analysis due to its small sample size. A re-analysis of infection rates between Henan and Shaanxi Provinces was then conducted, revealing a statistically significant difference in prevalence between the two provinces (*χ*^2^ = 10.139, *p* = 0.001). Additionally, *E. bieneusi* was detected in 13 of 34 pig farms (Fig. 1), with a farm-level positivity rate of 38.2% (95% CI, 21.0–55.4).

**Table 2:** Risk factors associated with the prevalence of *Enterocytozoon bieneusi* among rodents and shrews inhabiting pig farms.

Variable		No. positive/No. tested	Positive rate % (95% CI)	Univariate analysis		Multivariate analysis	
				*p-*value	OR (95% CI)	*p-*value	OR (95% CI)
Host Species	*Rattus tanezumi*	32/136	23.5 (16.3–30.7)	0.018	3.54 (0.79–15.83)	0.259	4.01 (0.75–21.62)
	*Rattus norvegicus*	5/58	8.6 (1.2–16.1)		1.085 (0.20–6.01)		2.02 (0.29–14.01)
	*Mus musculus*	2/25	8.0 (−3.4–19.4)		1		1
	Others	0/8	0 (−)		na		na
Host Age	Juvenile	11/79	13.9 (6.1–21.7)	0.342	1		
	Adult	28/148	18.9 (12.5–25.3)		1.44 (0.68–3.08)		
Host Sex	Female	17/110	15.5 (8.6–22.3)	0.504	1		
	Male	22/117	18.8 (11.6–26.0)		1.27 (0.63–2.54)		
Region	Henan	25/189	13.2 (8.4–18.1)	0.002	1	0.006	1
	Shaanxi	12/34	35.3 (18.4–52.2)		3.58 (1.58–8.12)		3.46 (0.46–26.30)
	Shanxi	2/4	50.0 (−41.9–141.9)		6.56 (0.88–48.70)		4.01 (0.75–21.62)
Season	Spring	25/135	18.5 (11.9–25.2)	0.551	1		
	Summer	11/54	20.4 (9.3–31.5)		1.13 (0.51–2.49)		
	Autumn	3/9	33.3 (−5.1–71.8)		2.20 (0.52–9.40)		
	Winter	0/29	0 (−)		na		
Farm type	Breeding farm	30/166	18.1 (12.2–24.0)	0.557	1.28 (0.57–2.87)		
	Fattening farm	9/61	14.8 (5.6–23.9)		1		
Farm size	＞10000	14/99	14.1 (7.2–21.1)	0.105	1	0.167	1
	2000 ~ 10000	25/109	22.9 (14.9–31.0)		1.81 (0.88–3.71)		2.35 (0.97–5.67)
	<2000	0/19	0 (−)		na		na
Duration of pig rearing	>5 years	13/78	16.7 (8.2–25.1)	0.537	1.11 (0.50–2.47)		
	3–5 years	16/105	15.2 (8.2–22.2)		1		
	<3 years	10/44	22.7 (9.8–35.6)		1.64 (0.68–3.96)		

OR, odds ratio; CI, confidence interval; na, not applicable.

### Risk factors of *Enterocytozoon bieneusi* infection

Univariate analysis demonstrated a connection between *E. bieneusi* infection and region and host species (Table 2). However, factors such as host age, host sex, season, farm type, farm size, and duration of pig rearing did not influence *E. bieneusi* infection. Further analysis using the variables (farm size, region, and host species) included in the multivariate model identified regional factors (*p* = 0.006) significantly associated with *E. bieneusi* infection, which indicates an increased risk of rodents with *E. bieneusi* infection on pig farms in Shaanxi (OR = 3.46, 95% CI: 0.46–26.30) and Shanxi (OR = 4.01, 95% CI: 0.75–21.62) provinces relative to the Henan region (Table 2).

### Characterization and distribution of the *Enterocytozoon bieneusi* genotypes

**Figure 2:**
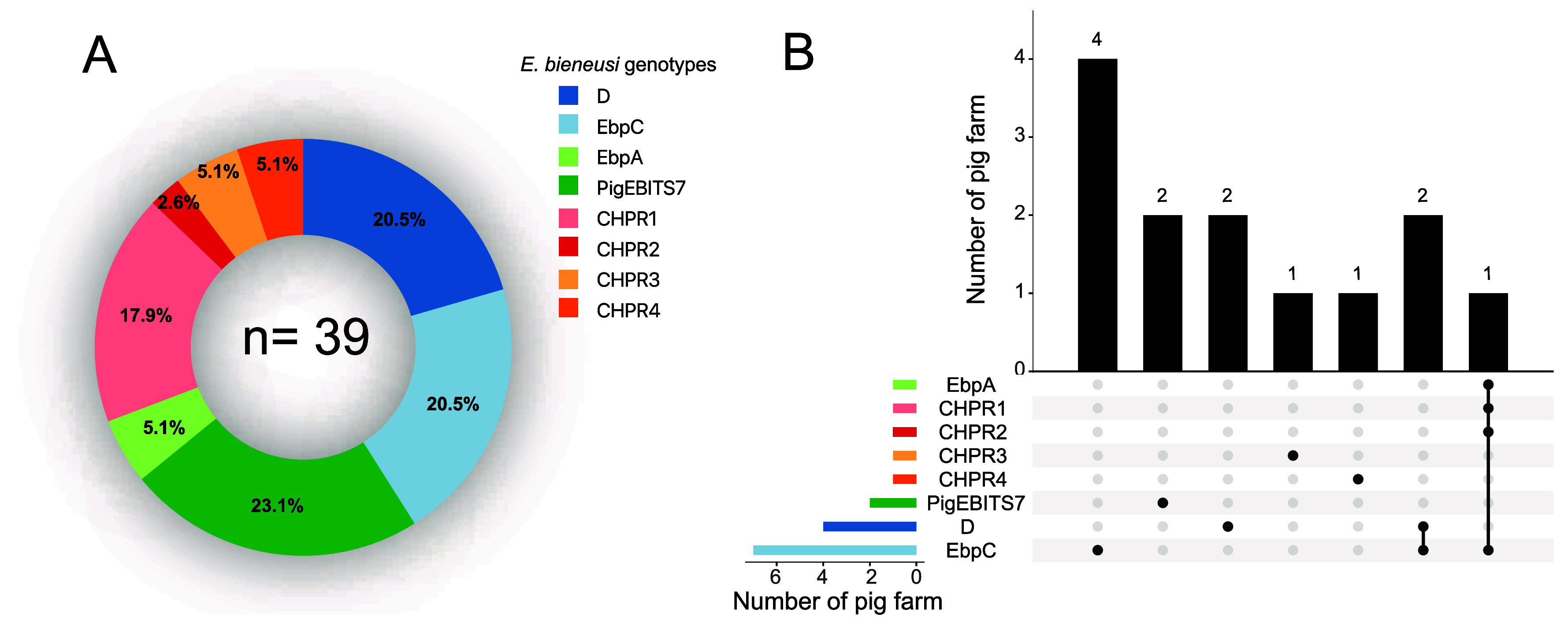
Prevalence rates and frequency of *Enterocytozoon bieneusi* genotypes in rodents and shrews on pig farms. (A) Prevalence rates of *E. bieneusi* genotypes in this study. (B) Frequency of *E. bieneusi* genotypes on pig farms.

Eight distinct genotypes of *E. bieneusi* were identified from the ITS sequencing of 39 isolates. These comprised four known genotypes (D, EbpC, PigEBITS7, and EbpA) and four newly named genotypes (CHPR1, CHPR2, CHPR3, and CHPR4). The most prevalent genotype was PigEBITS7 (23.1%, 9/39), followed by D and EbpC (20.5%, 8/39). CHPR1 accounted for 17.9% (6/39) of the isolates, while EbpA, CHPR3, and CHPR5 each represented 5.1% (2/39). CHPR2 exhibited the lowest prevalence at 2.6% (1/39) (Table 1 and Fig. 2A). Interestingly, although the PigEBITS7 genotype was predominant, the EbpC genotype had the broadest distribution and was present on seven pig farms that tested positive for *E. bieneusi* (Fig. 1 and Fig. 2B).

Distinct patterns of *E. bieneusi* genotype distributions were observed among different rodent species (Table 1). In all, three rodent species that tested positive for *E. bieneusi*, Genotype EbpC were detected. Genotype D was detected in both *R. tanezumi* and *R. norvegicus*. Genotypes EbpA, PigEBITS7, and the newly identified genotypes (CHPR1–CHPR4) were exclusively observed in *R. tanezumi*.

Nucleotide sequence analysis revealed that the new genotypes CHPR1, CHPR2, and CHPR3 exhibited base substitutions in the *E. bieneusi* ITS region (two, at positions 81 (C → T) and 95 (G → T); two, at positions 12 (G → A) and 196 (A → G); and one, at position 19 (G → A), respectively) in comparison to the EbpC genotype from a wild boar sample (GenBank accession number MK681466). CHPR4 displayed a single base substitution at position 118 (G → A) in the *E. bieneusi* ITS region, aligning with genotype H (GenBank accession number AF135835) from a pig sample source (Table 3).

**Table 3:** Nucleotide variations in the ITS gene region of newly identified *Enterocytozoon bieneusi* isolates in this study.

Genotype	GenBank ID	Nucleotide at position (ITS)					
		12	19	81	95	118	196
Novel							
CHPR1	PP158575	G	G	T	T	G	A
CHPR2	PP158576	A	G	C	G	G	G
CHPR3	PP158577	G	A	C	G	G	A
CHPR4	PP158574	G	G	T	T	A	G
Known							
H	AF135835	G	G	T	T	G	G
EbpA	OQ520128	G	G	T	T	G	G
EbpC	MK681466	G	G	C	G	G	A

### Phylogenetic analysis of *Enterocytozoon bieneusi*

**Figure 3:**
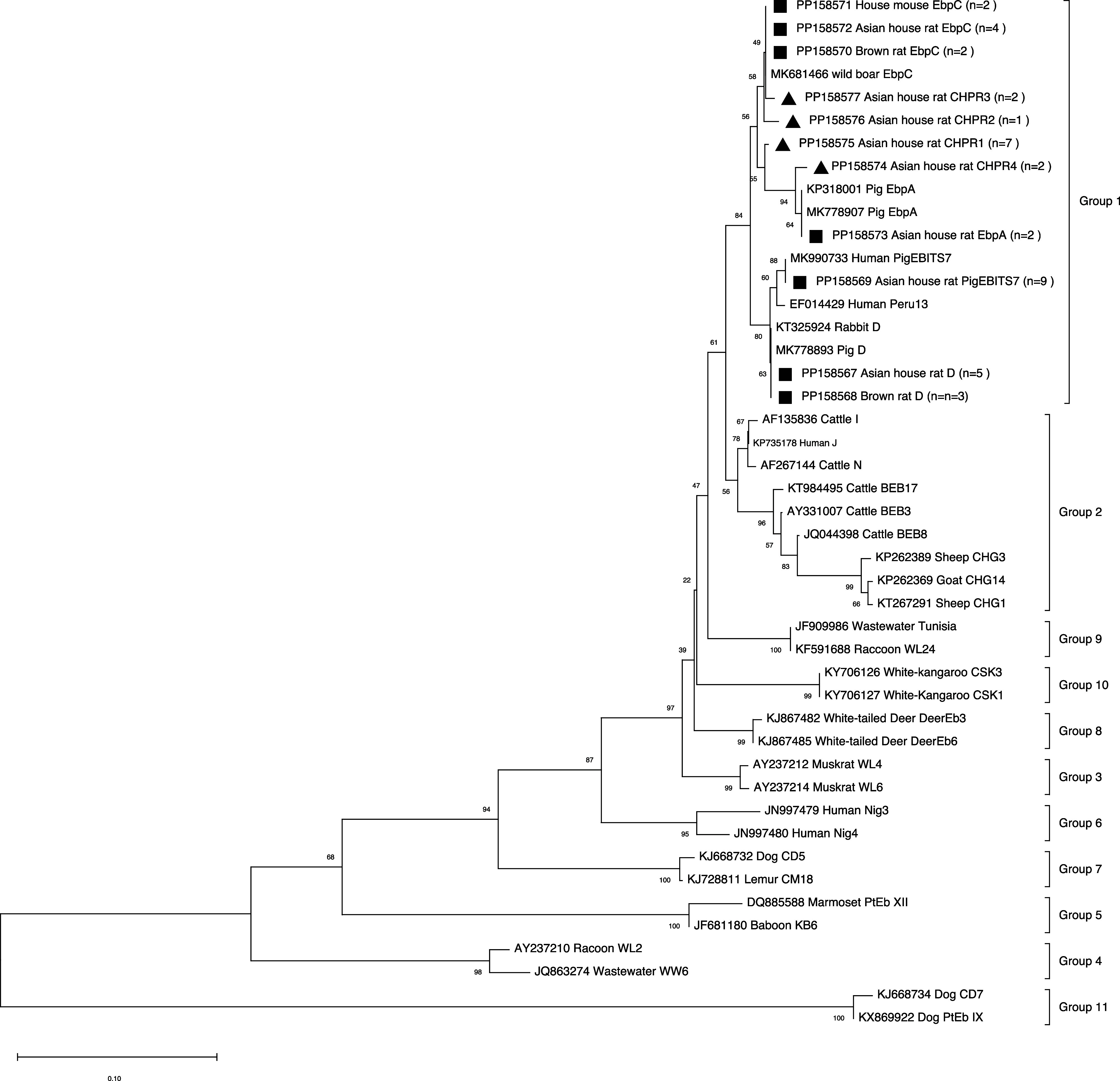
Phylogenetic relationships of *Enterocytozoon bieneusi* genotype isolates in this study. Based on sequence analysis of the ITS region, the neighbor-joining method and the Kimura-2 parameter model were used to analyze relationships. Known and novel genotypes are indicated by filled squares and triangles, respectively. Bootstrap values (>50) are indicated at the nodes.

A phylogenetic analysis of the *E. bieneusi* ITS region sequences revealed that all the genotypes obtained in this study were classified under group 1 (Fig. 3). The known genotypes PigEBITS7 and D were clustered in branch 1a; the new genotypes CHPR2 and CHPR3, and the known zoonotic genotype EbpC were clustered in branch 1d; and the new genotypes CHPR1 and CHPR4 and the known zoonotic genotype EbpA were clustered in branch 1e (Fig. 3).

## Discussion

*Enterocytozoon bieneusi* is a globally distributed opportunistic zoonotic pathogen [21]. Among the potential hosts of this pathogen, rodents play a significant role due to their frequent interaction with humans and domestic animals, as well as their wide presence in various environments [2, 33]. Despite the substantial research conducted on *E. bieneusi* infection in humans and domestic animals [35], there is limited information available regarding its prevalence in rodents, particularly in wild rodents inhabiting farm environments. Previous reports on *E. bieneusi* in rodents in farm settings have been scarce, with only one report each from a pig farm, cattle farm, sheep farm, and granary in Heilongjiang Province, as well as a cattle farm in Henan Province [54, 56]. Hence, this study provides the first report of *E. bieneusi* in rodents from pig farm environments in Henan, Shaanxi, and Shanxi Provinces of China.

Overall, 27 studies have reported the existence of *E. bieneusi* in rodents across eight different nations, with prevalence rates ranging from 1.1% to 100.0% (Table 4). It is noteworthy that, barring the cases of China and the USA, each country only underwent a single study. Therefore, these reported prevalence rates are likely to be theoretical rather than actual, emphasizing the need for additional comprehensive surveillance studies to ascertain the accuracy of these findings. Our research revealed an *E. bieneusi* infection prevalence of 17.2% (39/227), which was slightly higher than the worldwide prevalence of *E. bieneusi* infection in rodents (13.6%) [40]. However, this rate was significantly higher than the previously reported infection rate of 7.4% (19/242) for brown rats on a pig farm, cattle farm, sheep farm, and granary in Heilongjiang Province [56], as well as an infection rate of 4.0% (8/199) for brown rats and house mice on a cattle farm in Henan Province [54]. These differences in prevalence may be attributed to factors such as the environmental hygiene of the farms where the rodents were captured and the susceptibility of different rodent species to *E. bieneusi* [59]. Notably, pigs on farms with a high prevalence of *E. bieneusi* carriage are considered to be the main hosts of *E. bieneusi* [20, 23, 41], while rodents that forage freely on farms may acquire the infection through fecal contamination in intensive pig housing. The high field positivity rate (38.2%, 13/34) in our study appears to support this hypothesis. It is necessary to expand the survey and adopt a One Health approach to further investigate this discrepancy and determine the possible routes of transmission between farmed pigs and sympatric rodents.

**Table 4:** Prevalence and genotype distribution of *Enterocytozoon bieneusi* in rodents in different countries or areas.

Country	Rodent species	Feeding habits	No. positive/No. tested (%)	Genotype (n)	Reference
Iran	Brown rat (*Rattus norvegicus*)	Wild	13/146 (8.9)	D (11), M (2)	[42]
	Black rat (*Rattus rattus*)	Wild	1/14 (7.1)	D (1)	[42]
Japan	Pallas’s squirrel (*Callosciurus erythraeus*)	Wild	55/423 (13.0)	SCC-2 (55)	[27]
Peru	Guinea pig (*Cavia porcellus*)	Pet	10/67 (14.9)	Peru16 (10)	[4]
Poland	Striped field mouse (*Apodemus agrarius*)	Wild	79/184 (42.9)	D (6), WR5 (1), WR7 (1), gorilla 1 (1), WR8 (2)	[31]
	Yellow-necked mouse (*Apodemus flavicollis*)	Wild	18/60 (30.0)	WR4 (1), WR6 (6), WR1 (1), WR9 (1), D (2)	[31]
	Bank vole (*Myodes glareolus*)	Wild	18/46 (39.1)	WR6 (2), WR10 (2), WR2 (1), D (2),	[31]
	House mice (*Mus musculus*)	Wild	6/21 (28.6)	WR3 (1)	[31]
Slovakia	House mice (*Mus musculus*)	Wild	3/280 (1.1)	Unknown (3)	[6]
Czech-Germany border	House mice (*Mus musculus*)	Wild	31/289 (10.7)	EpbA (2), D (10), PigEBITS5 (7), C (2), H (1), CZ3 (4), Peru 8 (4), S6 (1)	[36]
USA	Black-tailed prairie dog (*Cynomys ludovicianus*)	Farmed	14/29 (48.3)	Row (14)	[34]
	Beaver (*Castor canadensis*)	Wild	13/85 (15.3)	WL7 (1), WL9 (1), WL12 (1), WL15 (1), WL8 (4), WL13 (5)	[39]
	Muskrat (*Ondatra zibethicus*)	Wild	20/239 (8.4)	WL4 (10), WL5 (1), WL6 (1), WL8 (2), WL10 (1), WL14 (1), WL15 (1), WL16 (3)	[39]
	Eastern gray squirrel (*Sciurus carolinensis*)	Wild	11/34 (32.4)	Type IV (3), WL4 (5), WW6 (2), PtEbV + WL21 (1)	[13]
	Eastern chipmunk (*Tamias striatus*)	Wild	5/7 (71.4)	Type IV (1), WL4 (3), WL23 (1)	[13]
	Woodchuck (*Marmota monax*)	Wild	5/5 (100.0)	Type IV + WL20 (1), WL4 (2), WL22 (1), WW6 (1)	[13]
	Deer mouse (*Peromyscus sp.*)	Wild	13/55 (23.6)	WL4 (10), WL23 (2), WL25 (1)	[13]
	Boreal red-backed vole (*Myodes gapperi*)	Wild	1/5 (20.0)	WL20 + WL21 (1)	[13]
	Meadow vole (*Microtus pennsylvanicus*)	Wild	3/10 (30.0)	Peru11 (1), Peru11 +type IV (1), WL21 + unknown (1)	[13]
China	Red squirrel (*Sciurus vulgaris*)	Pet	61/314 (19.4)	D (27), SCC-2 (18), SCC-3 (12), RS01 (2), RS02 (2)	[8]
	Edwards’s long-tailed giant rat (*Leopoldamys edwardsi*)	Wild	39/111 (35.1)	D (14), K (8), PigEBITS7 (22), Peru8 (2), GDR-2 (1), GDR-1 (10), CQR-2 (15), CQR-3 (1), GDR-3 (1), GDR-1 (2)	[12]
	Bower’s white-toothed rat (*Berylmys bowersi*)	Wild	37/117 (31.6)		[12]
	Brown rat (*Rattus norvegicus*)	Wild	19/242 (7.9)	D (17); Peru6 (2)	[56]
	Laboratory rat	Lab	14/291 (4.8)	EbpA (7), EbpC (3), CHY1 (2), N (1), SHR1 (1)	[18]
	Fancy rats (*Rattus norvegicus domestica*)	Pet	17/152 (11.2)	D (12), Peru11 (3), S7 (1), SCC-2 (1)	[45]
	Guinea pig (*Cavia porcellus*)	Pet	35/173 (20.2)	S7 (30), PGP (5)	[45]
	Himalayan marmots (*Marmota himalayana*)	Wild	47/399 (11.8)	YAK1 (17), ZY37 (27), SN45 (1), XH47 (1), ZY83 (1)	[51]
	Alashan ground squirrels (*Spermophilus alashanicus*)	Wild	3/99 (3.0)	HN39 (1), HN96 (1), YAK1 (1)	[51]
	Asian house rat (*Rattus tanezumi*)	Wild	31/134 (23.1)	PigEbITS7 (16); D (12); ESH-02 (1); Type-IV (1); EbpA (1)	[59]
	Brown rat (*Rattus norvegicus*)	Wild	8/56 (14.3)	D (3); PigEbITS7 (1); Type IV (1); Peru 8 (1); HNR-I (1); HNR-II (1)	[59]
	Edwards’s long-tailed giant rat (*Leopoldamys edwardsi*)	Wild	3/38 (7.9)	D (2); HNR-III (1)	[59]
	Chinese white-bellied rat (*Niviventer confucianus*)	Wild	6/33 (18.2)	D (3); PigEBITS7 (2); Type-IV (1)	[59]
	Indo-Chinese Forest rat (*Rattus andamanensis*)	Wild	5/54 (9.3)	D (3); Type-IV (1); HNR-III (1)	[59]
	Lesser rice-field rat (*Rattus losea*)	Wild	16/44 (36.4)	HNR-VII (15); D (1)	[59]
	Red-bellied squirrel (*Callosciurus erythraeus*)	Wild	1/24 (4.2)	D (1)	[59]
	Asiatic brush-tailed porcupine (*Atherurus macrourus*)	Farmed	7/93 (7.5)	D (3); HNR-VI (2); S7 (1); CHG5 (1)	[59]
	Bamboo rat (Rhizomyidae)	Farmed	18/117 (15.4)	D (15); Peru 11 (1); HNR-IV (1); HNR-V (1)	[59]
	Rodents (*Rattus norvegicus* + *Mus musculus*)	Wild	8/99 (4.0)	CHG14 (3), BEB6 (2), D (2), CHG2 (1)	[54]
	Chipmunks (*Eutamias asiaticus*)	Farmed	13/103 (12.6)	D (3); Nig 7 (2); CHY1 (3); SCC-1 (4); SCC-4 (1)	[10]
	Chipmunks (*Eutamias asiaticus*)	Pet	36/176 (20.5)	D (3); Nig 7 (2); CHG9 (2); CHY1 (2); SCC-1 (13); SCC-2 (9); SCC-3 (5);	[10]
	Red-bellied Tree squirrel (*Callosciurus erythraeus*)	Farmed	8/49 (16.3)	D (7), CE02 (1)	[9]
	Red-bellied Tree squirrel (*Callosciurus erythraeus*)	Pet	16/95 (16.8)	D (11), EbpC (3), SC02 (1) CE01 (1),	[9]
	Long-tailed chinchilla (*Chinchilla lanigera*)	Farmed	3/58 (5.2)	BEB6 (1), D (2)	[32]
	Long-tailed chinchilla (*Chinchilla lanigera*)	Pet	2/82 (2.4)	BEB6 (2)	[32]
	Coypu (*Myocastor coypus*)	Farmed	127/308 (41.2)	CHN4 (111), EbpA (7), EbpC (8), CNCP1 (1)	[52]
	Laboratory rat	Lab	9/118 (7.6)	J (2), BEB6 (7)	[48]
	Laboratory mice	Lab	18/1027 (1.8)	D (1), J (4), BEB6 (6), CHG10 (1), Henan-IV (4), WL6 (2)	[48]
	Laboratory guinea pigs	Lab	10/92 (10.9)	S7 (10)	[48]
	Hairless guinea pigs (*Cavia porcellus*)	Pet	6/35 (17.1)	S7 (5), PGP (1)	[25]
	Hairless guinea pigs (*Cavia porcellus*)	Farmed	40/289 (13.8)	S7 (37), PGP (3)	[25]
	Golden hamsters (*Mesocricetus auratus*)	Pet	26/175 (14.9)	D (23), Henan-II (1), SHW5 (1), Ebph1 (1)	[26]
	Siberian hamsters (*Phodopus sungorus*)	Pet	16/175 (9.1)	Ebph2 (9), D (4), Ebph3 (1), Ebph4 (1), Ebph5 (1)	[26]
	Brown rat (*Rattus norvegicus*)	Wild	53/399 (13.3)	EbpA (6), EbpC (19), D (17), ZJR1 (1), ZJR2 (1), ZJR3 (1), ZJR4 (1), ZJR5 (1), ZJR6 (1), ZJR7 (2), XJP-II (1), NCF2 (1), SDR1 (1)	[29]
	House mice (*Mus musculus*)	Wild	5/74 (6.8)	GXR1 (5)	[29]
	Daurian ground squirrel (*Spermophilus dauricus*)	Wild	4/41 (9.8)	EbpC (1), HLJC1 (2), HLJC2 (1)	[29]
	Plateau zokor (*Myospalax baileyi*)	Wild	4/98 (4.1)	QH219-5, QH219-9, QH219-13, QH219-21	[14]
	Asiatic brush-tailed porcupines (*Atherurus macrourus*)	Farmed	24/164 (14.6)	D (2), EbpD (2), EbpA (1), EbpC (1), ETMK5 (7), HNR-VII (1), HNHZ-I to HNHZ-IV (one each), S7 (1), TypeIV (4) HNPL-III (1),	[57]
	Bamboo rat (*Rhizomys sinensis*)	Farmed	128/303 (42.2)	D (76), Henan-III (21), HNZS-I (1), KIN-1 (11), SHW7 (19)	[57]
	Bamboo rat (*Rhizomys sinensis*)	Farmed	22/435 (5.1)	D (17), J (1), BR1 (1), BR2 (1), EbpA (1), PigEBITS7 (1)	[44]

To date, *E. bieneusi* infections have been reported in approximately 38 rodent species (Table 4). In this study, we investigated a total of five rodent and shrew species in piggeries and found *E. bieneusi* infections in the three most common rodent species (*R. tanezumi*, *R. norvegicus*, and *M. musculus*). We observed significant differences in prevalence rates among these species, suggesting variations in susceptibility to *E. bieneusi* (Table 2).

*Rattus tanezumi*, which is one of the dominant species of house rats in China [16], exhibited the highest prevalence of infection at 23.53% (32/136) in our study. Although this prevalence was significantly greater than the global infection rate of *E. bieneusi* in rodents (13.6%) [40], it closely matched the previously reported prevalence rate of wild *R. tanezumi* in Hainan Province, China (23.1%, 31/134) [59], indicating a high susceptibility of *R. tanezumi* to *E. bieneusi*. *Rattus norvegicus*, one of the most common commensal rodent species in the world and dominant in China [16], had an infection prevalence rate of 8.62% (5/58) in our study, which was lower than the prevalence rates observed in wild *R. norvegicus* in Hainan Province (14.3%, 8/56) [59] and in wild *R. norvegicus* in six provinces of China (13.3%, 53/399) [29], but similar to the prevalence rates found in wild *R. norvegicus* from Heilongjiang Province of China (7.9%, 19/242) and wild *R. norvegicus* from Iran (8.9%, 13/146) [42, 56]. Similarly, *Mus musculus*, one of the world’s most common commensal rodent species with a wide distribution in China [53], exhibited an infection prevalence rate of 8.00% (2/25) in our study. This rate fell within the range of reported global *E. bieneusi* infection rates in *M. musculus* (1.1–28.6%) [6, 29, 31, 36], but was higher than the prevalence observed in wild *M. musculus* in Slovakia (1.1%, 3/280) and in wild *M. musculus* from six provinces in China (6.8%, 5/74) [6, 29] and lower than the prevalence observed in wild *M. musculus* from Germany and the Czech Republic (10.7%, 31/289) and from Poland (28.6%, 6/21) [31, 36]. However, *E. bieneusi* was not detected in sporadically sampled individuals of *A. agrarius* (*n* = 1) or *C. shantungensis* (*n* = 7) in our study. Globally, there have been no reports of *E. bieneusi* infecting *C. shantungensis*, and the only analysis of *E. bieneusi* in *Apodemus agrarius* was carried out in Poland, with a prevalence rate of 42.9% (79/184) [31]. The infection rates of the rodent *E. bieneusi* show substantial differences in previous studies, potentially affected by factors like host immunity, sample size, experimental methodology, climate, and geographic differences. Therefore, further research is required to identify the factors contributing to this variation.

Table 4 demonstrates that rodents worldwide have been documented with nearly 70 different genotypes of *E. bieneusi*. In this study, eight genotypes of *E. bieneusi* were identified, including the zoonotic genotypes D, EbpC, PigEBITS7, and EbpA [20, 23]. The predominant PigEBITS7 genotype was exclusively found in *R. tanezumi* from two farms, accounting for 23.1% (9/39) of the *E. bieneusi* isolates. Originally found in pigs in Massachusetts, USA, it was subsequently identified in immunocompromized patients in Jiangxi and Henan in China, as well as in monkeys, wild rats, farmed bamboo rats, and wastewater in China [3, 20, 24, 47, 58]. Notably, this particular genotype has not been found in farmed pigs in China. Therefore, rodents infected with this genotype may be more closely associated with humans and other animal hosts on farms, but still pose a potential risk of infection in farmed pigs. Genotype D, which accounted for 20.5% (8/39) of the *E. bieneusi* isolates, was found in *R. tanezumi* and *R. norvegicus* from four pig farms. This genotype, widely prevalent in rodents, has also been linked to human infections in over 20 countries and has been isolated from more than 25 domestic and wildlife animals, as well as water sources, making it one of the most common *E. bieneusi* genotypes with a high risk of zoonotic transmission [20, 23, 35, 41, 59].

Genotype EbpC was detected in *R. tanezumi*, *R. norvegicus*, and *M. musculus* from seven pig farms, representing 20.5% (8/39) of the *E. bieneusi* isolates. As far as we are aware, this is the first documentation of this genotype being identified in *M. musculus* and *R. tanezumi*. Notably, this genotype is commonly found in humans, domestic and wild animals, and water sources worldwide [20, 23, 35]. Interestingly, it has been identified as the most prevalent zoonotic genotype in farmed pigs in China [41, 49] and exhibits the broadest distribution in our study area. Additionally, a survey on porcine intestinal parasites conducted on pig farms within our sampling area revealed a 9.5% detection rate of *E. bieneusi*, with 133 out of 1402 pig fecal samples collected testing positive. Among the positive samples, 54.1% (72 out of 133) had the EbpC genotype (unpublished data). We therefore hypothesized that this finding provides potential evidence of cross-species transmission between farmed pigs and sympatric rodents, but the possibility of other routes of transmission should also be considered, e.g., the possibility that pigs and rodents in the same area may share contaminated food, water, and environment, which needs to be confirmed by further investigation.

Genotype EbpA has been detected in various rodent species worldwide, including *R. norvegicus*, *R. tanezumi*, *M. musculus*, *Myocastor coypus*, *Atherurus macrourus*, and *Rhizomys sinensis* (Table 4) [29, 36, 44, 53, 59]. In our study, it was exclusively identified in *R. tanezumi* from one pig farm, accounting for 5.1% (2/39) of the *E. bieneusi* isolates. Moreover, this genotype has been observed in a wide range of hosts, including humans, nonhuman primates, domestic animals (cattle, buffalo, horses, sheep, and goats), pets (dogs), wildlife (deer, foxes, raccoons, bears, pandas, and otters), and birds (pigeons, cranes, and parrots) [20, 23, 28, 37]. It has also been detected in river water and wastewater treatment plants [15, 19, 35, 52]. Thus, the potential cross-species transmission of genotype EbpA poses a zoonotic risk to humans and other animals, while sympatric rodents may act as reservoir hosts for EbpA during the transmission of *E. bieneusi*.

Four new genotypes of *E. bieneusi* were identified in this study through sequence analysis of the ITS regions of the obtained *E. bieneusi* isolates. These novel genotypes were classified into group 1 through phylogenetic analysis and showed one or two base substitutions compared with known zoonotic genotypes, indicating a high risk of zoonotic transmission [20]. Consequently, sympatric rodents infected with *E. bieneusi* could potentially serve as reservoirs of transmission to humans and other animals, posing a potential threat to public health and ecological security. Additionally, given that farmed pigs are the main hosts of human pathogenic *E. bieneusi* [20, 23, 41], sympatric rodents inhabiting pig farm environments may facilitate the spillover transmission of this pathogen from pig farms. This is because these rodents are common shuttles between human and animal hosts, as well as between domestic and natural environments. Therefore, controlling rodent populations in the surveyed areas and raising awareness among local populations about the risk of transmission of *E. bieneusi* from rodents to humans are necessary measures to reduce the threat to public health.

## Conclusions

*Enterocytozoon bieneusi* infection is prevalent in wild sympatric rodents inhabiting pig farm environments in China, with the zoonotic genotype EbpC showing the broadest distribution. The study identified all genotypes as belonging to group 1, indicating that sympatric rodents serve as natural reservoirs for *E. bieneusi*, posing a potential risk to public health and ecological stability. These findings provide possible evidence for cross-species transmission between sympatric rodents and domestic pigs, implying that sympatric rodents may facilitate the spillover of *E. bieneusi* from pig farms. Further molecular epidemiological investigations using a One Health approach should be conducted to assess the role of these sympatric rodents in *E. bieneusi* transmission and explore possible transmission routes in the survey area.
